# Low-coverage whole-genome sequencing of extracellular vesicle-associated DNA in patients with metastatic cancer

**DOI:** 10.1038/s41598-021-83436-1

**Published:** 2021-02-17

**Authors:** Bella Nguyen, Nicholas C. Wong, Tim Semple, Michael Clark, Stephen Q. Wong, Connull Leslie, Bob Mirzai, Michael Millward, Katie Meehan, Annette M. Lim

**Affiliations:** 1 Department of Medical Oncology, Sir Charles Gairdner Hospital, Perth, WA Australia; 2 School of Biomedical Sciences, University of Western Australia, Perth, WA Australia; 3 Monash Bioinformatics Platform, Monash University, Victoria, Australia; 4 Department of Medical Oncology, Peter MacCallum Cancer Centre, Melbourne, VIC Australia; 5 Peter MacCallum Cancer Centre, Melbourne, VIC Australia; 6 School of Medical and Health Sciences, Edith Cowan University, Joondalup, WA Australia; 7 School of Pathology and Laboratory Medicine, The University of Western Australia, Perth, WA Australia; 8 Department of Anatomical Pathology, PathWest, QEII Medical Centre, Perth, WA Australia; 9 Department of Otorhinolaryngology, Head and Neck Surgery, Chinese University of Hong Kong, Shatin, Hong Kong; 10 School of Medicine, The University of Western Australia, Perth, WA Australia

**Keywords:** Head and neck cancer, Tumour biomarkers

## Abstract

Low-coverage whole-genome sequencing (LC-WGS) can provide insight into oncogenic molecular changes. Serum extracellular vesicles (EV) represent a novel liquid biopsy source of tumoral DNA. This study compared copy number alteration (CNA) profiles generated from LC-WGS of formalin-fixed paraffin-embedded (FFPE) tumoral DNA and EV-DNA obtained from cancer patients. Patients with squamous cell carcinoma of the base of tongue (n = 3) and cutaneous squamous cell carcinoma (n = 2) were included. LC-WGS (0.5-1X coverage) was performed on FFPE-DNA and serum EV-DNA. Similarity between CNA profiles was analysed using QDNAseq. FFPE samples had a mean CNA of 31 (range 17–50) over 1.9 × 10^9^ (range 1.0–2.6 × 10^9^) bp in length, and EV samples had a mean CNA value of 17 (range 7–19) over 7.6 × 10^8^ (range 2.9–15 × 10^8^) bp in length. A mean of 8 (range 0–21) CNA over 5.9 × 10^8^ (range 1.6–14 × 10^8^) bp in length was found to overlap between EV and FFPE-derived samples per patient. Although the mean correlation efficient between samples was *r* = 0.34 (range − .08 to 0.99), this was not statistically significant (*p* > 0.05). Regions of highest deletion and duplication in FFPE samples were not well reflected in the EV-DNA. Selected CNA regions in EV-associated DNA were reflective of the primary tumor, however appreciation of global CNA and areas of most significant change was lost. The utility of LC-WGS of EV-derived DNA is likely limited to molecular alterations of known interest.

## Introduction

Liquid biopsies facilitate the non-invasive characterization of tumor-derived molecular contents from biofluids. They represent a new frontier for personalized medicine approaches and can provide real-time tumor assessments that can guide treatment. Beyond cell-free DNA (cf-DNA), extracellular vesicles (EV) are emerging as a novel and complementary source of tumor-derived DNA. EV are a heterogenous group of particles with a phospholipid bilayer membrane, found abundantly in various biofluids such as blood, urine, saliva and cerebrospinal fluid^1^. They are recognized to be carriers of biologically active molecules, and participate in important biological functions including endocrine, paracrine, and autocrine signalling^1,2^. They are known to be associated with biofunctional proteins, RNA (including microRNAs), DNA, lipids, and other metabolic molecules^3^. Physiologically, EVs have also been implicated in specific biological processes such as neural plasticity, tissue repair, stem cell maintenance and blood coagulation^4^. EVs have been suggested to be involved in the pathogenesis of a wide range of disorders such as infections, cardiovascular disease and metabolic disorders^5–8^. In recent years, EVs have garnered significant interest as potential cancer biomarkers, as EV have been found to contain tumoral molecular content and are hypothesized to contain more intact elements that are actively secreted by dividing tumor cells compared to the more fragmented, apoptotic cellular materials derived from cell-free circulating nucleic acid^9–12^.

Copy number alteration (CNA) defined as a variable copy number of a segment of deoxyribonucleotides > 1 kb in size is a commonly observed major genetic alteration identified in most cancers^13–16^, that has been postulated to be both a predictive and prognostic biomarker^14,15^. As cancer genomes are known to evolve over time CNA can also be observed to fluctuate, and CNA can also arise due to treatment response or resistance^14^. Therefore, DNA extracted from circulating EV from cancer patients’ biofluids can facilitate a convenient and non-invasive continual assessment of CNA changes in a tumor during treatment, and can provide insight into the mechanisms behind disease progression. There are a number of approaches currently available to assess CNA in formalin-fixed paraffin-embedded (FFPE) tumoral tissues including microarray-based comparative genomic hybridization (array-CGH), single nucleotide polymorphism (SNP) arrays, molecular inversion probe (MIP) assays, and low coverage whole genome sequencing (LC-WGS)^16,17^. Traditionally CNA is assessed using high yields of DNA obtained from traditional tumoral biopsies, whilst EV-associated DNA is isolated in significantly smaller quantities. Kader et al. described a LC-WGS method which successfully characterized CNA using ultra-low input of tumoral FFPE-derived DNA^18^ and similarly, CNA has been demonstrated to be successfully profiled in smaller quantities of circulating-tumor DNA (ct-DNA) using LC-WGS^19^. Thus, LC-WGS could be used to assess EV-associated DNA.

This study aimed to assess whether LC-WGS of EV-associated DNA is reflective of tumoral DNA based on their respective CNA profiles, to define the potential clinical utility of this liquid biopsy approach. We compared CNA profiles generated from LC-WGS of DNA extracted from formalin-fixed paraffin-embedded (FFPE) samples and matched EV isolated from the serum of cancer patients.

## Results

### Demographic data

Demographic data of patients enrolled in the study are summarized in Table 1 (n = 5). Three patients had metastatic base of tongue squamous cell carcinoma (BOT), with two patients having Human Papilloma Virus (HPV)-related disease. Two patients had metastatic cutaneous squamous cell carcinoma (cSCC). Patients had a median age of 64 (range 59–77) years, and were predominantly male. Median follow up was 32 (range 22–41) months and at data cut-off, two patients had died of disease and three patients were alive and responding to treatment. Four patients had their EV samples collected for analysis within one month of their FFPE-tumor samples being obtained, and one patient (Study ID: C008) had their EV samples collected 12 months after their FFPE-tumor sample. All BOT patients have received radiation, chemotherapy and immunotherapy as treatment for their disease. All cSCC patients have received surgery and immunotherapy as treatment for their disease.

**Table 1:** Clinicopathological characteristics of the cohort.

Study ID	Age (years)	Sex	Tobacco use	Tumor	Clinical status	Follow up time (months)
B005	64	Male	Ex-smoker	mBOT SCC	Died from disease progression	35
				HPV +		
B010	62	Male	Never	mBOT SCC	Alive, responding to treatment	32
				HPV +		
B014	77	Female	Smoker	mBOT SCC	Alive, responding to treatment	41
				HPV −		
C004	70	Male	Ex-smoker	mcSCC	Alive, responding to treatment	24
C008	59	Female	Smoker	mcSCC	Died from disease progression	22

*mBOT SCC* metastatic base of tongue squamous cell carcinoma, *mcSCC* metastatic cutaneous squamous cell carcinoma.

### Validation of isolated EV & EV-DNA characteristics

**Figure 1:**
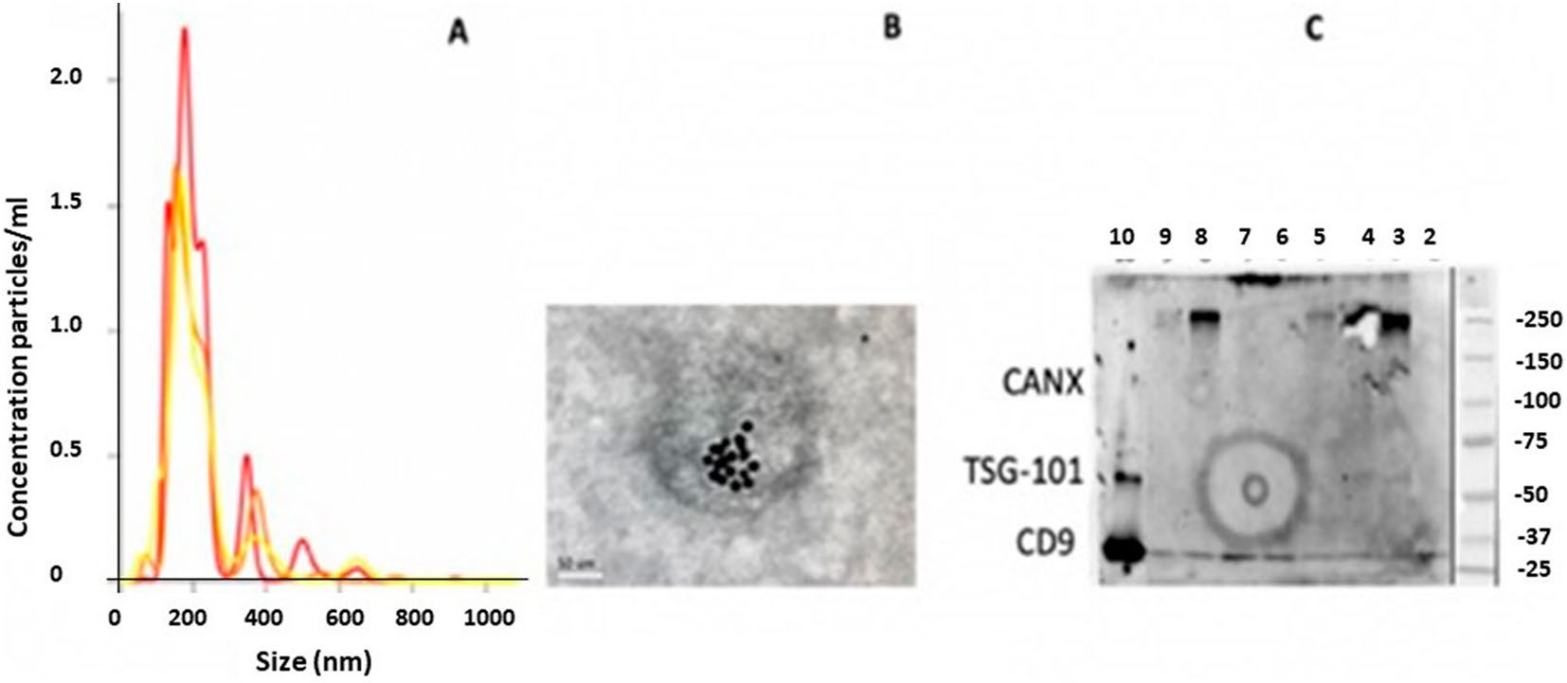
Validation and characterization of isolated EV: Size and concentration distribution of nanoparticles from NTA in triplicate (yellow, red and orange graphs) (**A**); ds-DNA antibody labelled TEM image at magnification × 40,000 (**B**); and Western blots using common exosome markers (positive for CD9 and negative marker Calnexin) in melanoma cell lysate (SKMel28) as positive control (lane 10) and isolated EV (lane 7) (**C**). Lanes 2, 3, 4, 5, 6, 8, 9 were of other samples that were included in the same Western Blot analysis, but were not patients included in the study.

Validation of EV isolation from differential ultracentrifugation (UC) was carried out using Transmission Electron Microscopy (TEM), Nanoparticle Tracking Analysis (NTA), and Western blotting with collective results presented in Fig. 1. Isolated EV analyzed in triplicate (Fig. 1A) by NTA show a mean particle size of 215 nm and mean concentration of 2.03 × 10^10^ particles/mL. Figure 1B shows a ds-DNA antibody labelled TEM image of a representative isolated EV, which demonstrates a round particle with a diameter of approximately 200 nm. Ds-DNA antibody positive particles are evident within the outline of the vesicular membrane. Figure 1C demonstrates the Western blot results using common exosomal markers (positive for CD9 and negative marker Calnexin) in melanoma cell lysate (SKMel28) as positive control (lane 10) and isolated EV (lane 7).

**Figure 2:**
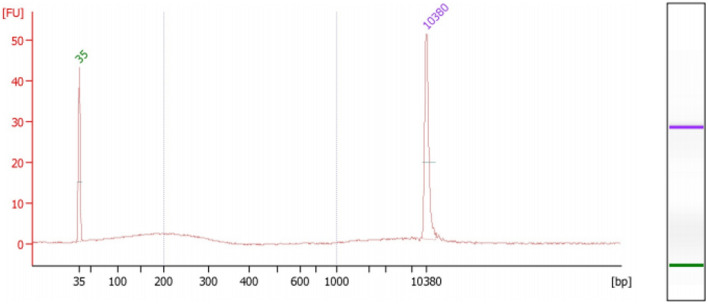
Sample extraction High Sensitivity DNA bioanalyzer trace for EV-associated DNA from patient #B-010.

A minimum of 10 ng of extracted EV-associated DNA was used for each LC-WGS run. One representative patient (#B-010) sample is shown in Fig. 2, which was analyzed using High Sensitivity DNA bioanalyzer (Agilent) to assess sample preparation, quality and fragment size of the EV-associated DNA extracted. The percentage of reads mapped from all patients’ EV-associated DNA ranged between 55.6 and 97.1% (mean = 71.6). Median fragment length (bp) ranged between 169 and 178 bp (mean = 176 bp). LC-WGS was performed aiming for 0.5–1 × coverage, and was considered adequate to generate CNA profiles.

### Comparison of CNA profiles of FFPE and EV samples

**Figure 3:**
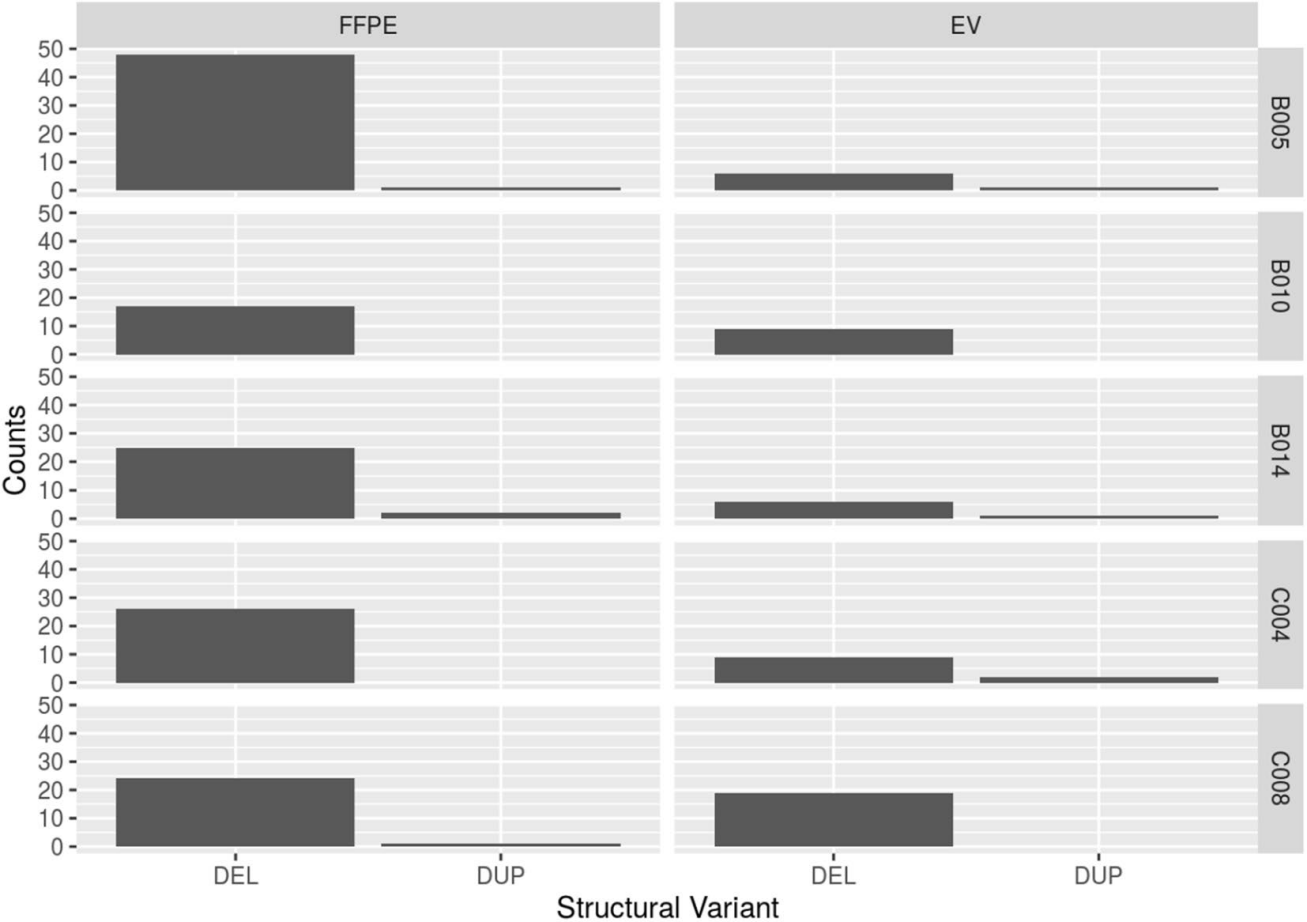
Comparison of CNA counts (deletion (DEL), or duplication (DUP)) of EV and FFPE samples, with patient ID labelled on the right of the figure. CNA counts are plotted on Y-axis. Segmentation analysis was performed using QDNASeq and the resultant output files were summarised using R, version 1.6.2^20^.

FFPE samples had a mean CNA of 31 (range 17–50) covering over 1.9 Gbp (range 1.0–2.6 Gbp) in length. EV samples had a mean CNA of 17 (range 7–19) covering 0.76 Gbp (range 0.29–1.5 Gbp) in length. The mean number of CNA and length of abnormal DNA that overlapped between both the FFPE samples and EV samples for the same patient for the whole cohort was 8 (range 0–21) CNA regions and 0.59 Gbp (range 0.16–1.4 Gbp) in length. Table 2 and Fig. 3 summarises the global CNA count (total number of deletions and duplications) for EV and FFPE samples of each patient, as well as areas with significant duplications and deletions. The majority of CNA in FFPE samples were deletions rather than duplications, and this was reflected in EV samples. However, the number of CNA identified in EV-DNA were considerably less than found in FFPE.

**Table 2:** CNA region summaries of FFPE/EV samples for each patient, with overlapped CNA regions (in both number of regions and number of basepairs), with correlation value (*r*) and *p* values.

Study ID	Tumor	CNA regions (number of region/basepair)		Overlapped CNA region (number of region/basepair)	Correlation value (*r* =)	Regions with significant deletion/duplication	
		FFPE	EV			FFPE	EV
B005	mBOT SCC HPV +	50 2.6 × 10^9^	16 6.2 × 10^8^	7 6 × 10^8^	− 0.49 (*p* = 0.31)	Deletion: 3p and 8p Duplication: 3q, 5p and 8q, 20p, 20q	Deletion: 15p Duplication: 7q, 14q and 15q
B010	mBOT SCC HPV +	17 1.1 × 10^9^	11 4.2 × 10^8^	2 1.6 × 10^8^	0.98 (*p* = 0.41)	Deletion: 1q Duplication: 8q, 11q, 20q	Deletion: 1q, 4p, 6q Duplication: 1q, 8q, 14q, 17q
B014	mBOT SCC HPV −	27 2.5 × 10^9^	7 2.9 × 10^8^	0 2.2 × 10^8^	− 0.08 (*p* = 0.48)	Deletion: 11q, 14q Duplication: 3q	Deletion: 15q Duplication: 5q14p
C004	mcSCC	30 1.7 × 10^9^	19 9.5 × 10^8^	11 5.1 × 10^8^	0.88 (*p* = 0.19)	Deletion: 8p Duplication: 7p, 8q, 20p	Deletion: 8p Duplication: 1p, 3p, 15q, 17q, 20q
C008	mcSCC	28 1.6 × 10^9^	31 1.5 × 10^9^	21 1.4 × 10^9^	0.42 (*p* = 0.86)	Deletion: 4q, 9p, 11q Duplication: 7p, 8q, 9q, 14q, 20p	Deletion: 2q, 4q, 6q, 8p, 11q Duplication: 5p, 14q, 15q

*mBOT SCC* metastatic base of tongue squamous cell carcinoma, *mcSCC* metastatic cutaneous squamous cell carcinoma.

Summarised in Table 2 are the regions of deletion/duplication in both number of CNA, and number of basepairs, the areas of overlapped CNA region, Pearsons correlation value (*r*) calculated of CNA in the overlapped regions, associated *p* values using paired sample t-test, and regions with significant (defined as high frequency) deletion/duplication between FFPE and EV samples of all patients included in the study. As noted in Fig. 3, EV samples reflected some specific CNA in FFPE but in general were less in number and some CNA were unique to EV samples suggesting the limitation of tumor sampling from both FFPE and EV. Despite the overlap, none of the correlation values were statistically significant with *p* values all greater than 0.05.

**Figure 4:**
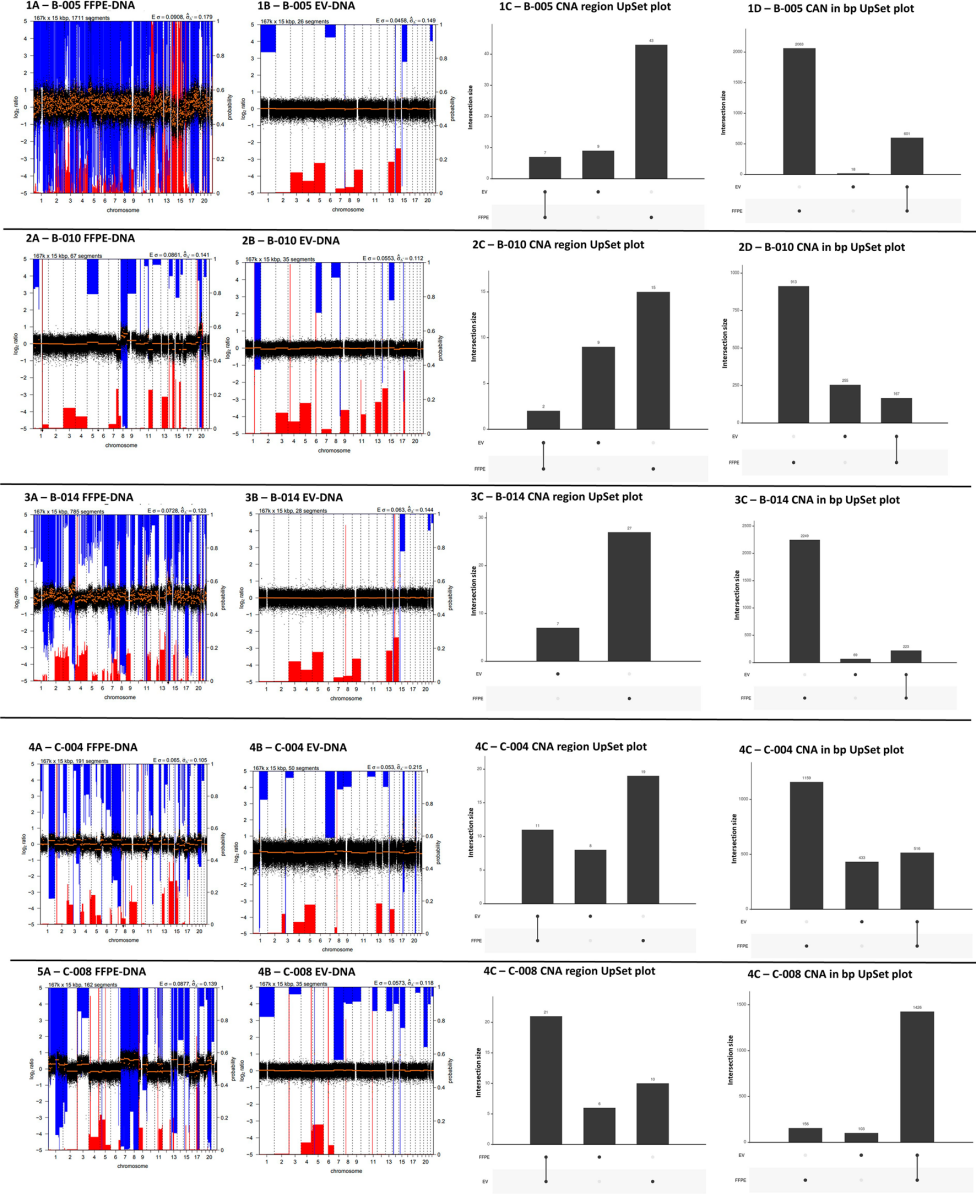
CNA profiles of all patients (#B-005, #B010, #B-014, #C-004, #C-008) included in the study FFPE tumors (1–5 **A**) and EV samples (1–5 **B**), with UpSet plots demonstrating overlapping regions between the sample sets for regions of CNA (1–5 **C**) and the length (million basepairs—1–5 **D**) of the altered regions identified. CNA profiles were determined using QDNAseq and the resultant output files were summarised using R, version 1.6.2^20^, with copy number loss (blue) and copy number gain (red) regions highlighted for all chromosomes. Frequency of CNA in log2 ratio are plotted on Y-axis, and chromosome coordinates from 1 to 22 are ploted on X-axis. UpSet plots demonstrate the number of events between samples as a column, with the sample type indicated beneath the plot and the total number of events annotated on top of the column. Concordance between samples is represented by the presence of a vertical connecting line between sample type beneath the X-axis.

The concordance of CNA profiles between matching FFPE tumors and EV samples are shown in Fig. 4. Overall, although common CNA and regions were identified between sample types, there were many areas under-represented by the EV-DNA based analyses. Again, some CNA were unique to each sample type demonstrating sampling limitations. In the HPV-driven metastatic BOT group (n = 2), patient #B-005 FFPE tumoral DNA demonstrated significant regions of deletion detected in Chr 3p and 8p, and duplication in Chr 3q, 5p and 8q, 20p and 20q. These observations are consistent with findings from The Cancer Genome Atlas (TCGA) head and neck cancer cohort and a large HPV-positive and HPV-negative oral squamous cell carcinomas data set published by Gillison et al.^21,22^. This patient’s profile also contained recurrent focal amplification for 3q26/28, a region involving squamous lineage transcription factors *TP63* and *SOX2* and the oncogene *PIK3CA*, which was also a group of mutations found in the TCGA dataset^21^. In contrast, patient #B-010 had a significant deletion observed in Chr 1q while regions with duplication were observed in Chr 8q, 11q and 20q. These findings are not observed in prior published datasets^21,22^. In the non HPV-driven metastatic base of tongue patient (#B-014), the only finding that is consistent with prior published dataset is in the duplication observed in Chr 3q. In the metastatic cutaneous squamous cell cancer samples, consistent with published literature were regions of duplication found in Chr 7p, 8q, and 20q seen in both FFPE and EV samples^23^.

## Discussion

LC-WGS is a rapid and practical approach to characterize large-scale chromosomal alterations across the genome, which can be used to monitor tumoral molecular evolution^14^. Non-invasive liquid biopsy approaches such as cf-DNA, ct-DNA or EV-associated DNA have emerged as viable alternatives to using traditional tumoral tissue which require an invasive biopsy. Studies applying LC-WGS using cf-DNA and ct-DNA analysis have demonstrated the ability to detect tumour-associated CNA changes in cancer patients with various cancer types including prostate cancer, colorectal cancer, lung cancer, urothelial cancer and neuroblastoma^24–31^. Changes in CNA profiles of cf/ct-DNA have also been to be associated clinical disease progress such as cancer proliferation, and treatment response^32,33^. However, LC-WGS is restricted by the use of samples in which the tumor fraction is relatively high (> 10%), thus the search for alternative sources of circulating DNA is critical^33^. A recent study found that EV-DNA contained a higher mitochondrial DNA copy number than that found in cf-DNA of patients with hepatocellular carcinoma using whole genome and capture-based sequencing, suggesting EV-DNA may be an advantageous source of alternative liquid biopsy^34^. Our study is one of the few published studies that has investigated the utility of LC-WGS as a method of CNA profiling using low-input EV-associated DNA from cancer patients’ plasma with comparison to matched FFPE samples.

EV have been reported to contain tumoral DNA elements such as highly expressed oncogenic gene mutations in several cancer models including melanoma, lung, pancreatic and colorectal cancer^9–11,35,36^. However, few studies have directly compared whole genome sequencing concordance between EV-associated DNA and the primary tumor. One study identified tumoral-related copy number variations (CNV) in 3 metastatic pancreatic patients’ EV-DNA^37^. Another study which has compared CNA profiles analysis using LC-WGS at 0.6X depth between EV-DNA and FFPE tumoral DNA was published by Lee^38^. This demonstrated that in nine urothelial bladder carcinoma patients, CNA analyses in EV-DNA and cf-DNA reported concordance with tumoral DNA (*r* = 0.412 and 0.481 respectively, *p* values not provided)^38^. Further, the authors were able to confirm that areas of CNA corresponded to regions with known mutations of interest including loci involving *CDKN2A* and *RB1*^38^*.*

Our study found that while overlapping regions of CNA in the EV samples and FFPE samples are observed, the correlation coefficients were highly variable (range: − 0.49 to 0.99) with no correlation being found to be statistically significant (*p* values > 0.05 from paired sample t-test). Regions in FFPE samples with significant duplication or deletion were not reflective of the regions found in EV samples, suggesting that EV-derived DNA probably represents a portion of tumoral DNA and non-tumoral DNA. Changes unique to EV-DNA could arise from non-tumoral DNA, mitochondrial DNA, or due to FFPE and EV sampling limitations. Overall there were significantly more regions of CNA detected in FFPE-DNA than in EV-DNA, likely due to its reported fragmented nature, which renders it difficult to detect larger CNA regions. These observations have identified some limitations of using CNA profiles generated from EV-associated DNA in the clinical setting.

It is difficult to make comparisons between studies, given the different cancer types, different methodologies used for EV isolation, and that global numbers of CNA and length were not reported in the discussed studies above. Our study also demonstrated that EV-DNA could identify CNA regions containing known cancer-relevant genes concordant with FFPE. However, we also showed that the global architecture of the molecular changes observed in FFPE and the regions of greatest change were lost with the limited view provided by the liquid biopsy approach. Specifically, we demonstrated that FFPE had a larger number of CNA spanning over a number of base pairs compared to EV-DNA and highest regions of loss or gains were observed in different regions between samples. EV-DNA can still potentially be used to identify specific mutations of known interest which was not pursued in depth within our study given the low coverage of the whole genome sequence. We recently reported that EV-DNA was able to detect circulating tumoral HPV-DNA in HPV-driven oropharyngeal carcinomas, however the detection sensitivity was significantly lower than cf-DNA analyses (*p* =  <0.001)^39^. One study using LC-WGS CNA profiles of cf-DNA reported good concordance with FFPE in 22 metastatic prostate cancer patients (*r* = 0.87, *p* < 0.001)^40^. It is however recognised that cf-DNA contains different DNA elements to EV-DNA and this may explain the difference reflected in the results^41^. Our findings calls for further research in comparing the clinical utility of EV-DNA to cf-DNA which is an already established, well validated source of liquid biopsy. One direction to improve similar studies in the future could be to use immunocapturing of specific EV subpopulations that are postulated to be involved in carcinogesis, thus increasing sensitivity of the EV-DNA analysis for the detection of tumour-derived DNA^42^. However, the ability to isolate circulating DNA that is tumour-specific remains a challenge unless a tumour is defined by the presence of known oncogene-addiction which is not the case for most malignancies.

There are limitations to this study. The study lacked the ability to standardize of amount of input EV-associated DNA used in LC-WGS due to limited yield which may have contributed to the variations in different CNA profiles. Due to the paucity of similar studies published in literature, an optimal amount of EV-DNA input has not yet been determined. We did not specifically select a specific EV subpopulation, which may alter EV-DNA yield and quality, with a recent study showing that large EVs (those > 1000 nm) contain more tumour-derived DNA than small EVs (< 200 nm)^43^. We also note that at our sequencing depth 0.5-1X and an average of 50 million reads (see “Methods and materials” section below), we may not have achieved the most optimal sequencing sensitivity of smaller tumour specific fragments. There are also limitations to using QDNASeq to perform CNA profiling, firstly the bioinformatic package cannot completely correct for technical bias in read coverage, and secondly, the copy number calling can still be impacted by DNA quality^44^. Additionally, our cohort of patients was small in number, included different tumor types and varied treatments. It has been demonstrated that different types of tumors with varying levels of genomic instability may result in different amounts of EV as well as diverse molecular packaging, and thus result in different types of DNA isolated^43^. EV samples were not collected at the same time as FFPE samples in our study as which utilised available archival FFPE samples similar to real life clinical practice or trial participation. Thus, the discordance of the two types of DNA CNA profiles may be impacted by the varied sample collection times. Finally, concordance of mutations would be more comprehensively assessed using orthogonal methods with deeper sequencing resolution or targeted sequencing which were not included in our study.

## Conclusion

Based on our results, we suggest that although EV-associated DNA CNA profiles were reflective of FFPE, the overlapping regions were limited in number, highly variable between patients, and did not globally reflect regions with significant alterations found in FFPE. Whilst EV-associated DNA may be useful as a liquid biopsy source to identify tumoral DNA, overall, they do not globally reflect primary tumor DNA CNA profiles. In a clinical setting, EV-associated DNA alone may not be suitable as a source to be used to monitor global tumoral DNA changes.

## Methods and materials

### Participants, ethics, consent and regulations

Five patients with metastatic cancer were recruited prospectively for the study between April 2016 and May 2018. All experimental protocols in the study received institutional approval from Sir Charles Gairdner Group Human Research Ethics Committee (HREC 2015-062). All experimental methods were carried out in accordance to the principles set out by the National Statement on Ethical Conduct in Human Research and Good Clinical Practice Guidelines. Study participants provided written informed consent prior to enrolment.

### Isolation and validation of EV

Isolation and validation of EV was carried out as per method previously published by our group^39^. Plasma collected in 4 × 10 mL EDTA tubes were processed within one hour. Cellular components were removed by centrifugation at 1600*g* for 20 min at room temperature. Samples were then centrifuged further at 14,000*g* for 10 min at room temperature to remove large particles such as platelets. Subsequently, clarified plasma (4000 µL) was diluted in PBS and centrifuged at 12,000*g* for 45 min, 110,000*g* for 90 min, and finally at 110,000*g* for 90 min at 4 °C using the Type 70 Ti rotor in the Optima L-90K Ultracentrifuge (Beckman Coulter, Australia). Validation of EV isolation was carried out according to guidelines recommended by the International Society of Extracellular Vesicles (ISEV) using Transmission Electron Microscopy (TEM), Nanoparticle Tracking Analysis (NTA), and Western blotting^45^. For TEM, EV were incubated with primary antibody (5 μL of mouse anti-human CD9, 10 μg/mL, Merck, Australia) and then with secondary antibody (5 μL of mouse anti-human IgG conjugate, LSBio, USA) on 200 mesh Formvar-carbon coated copper grids (ProSciTech, Australia). Grids were visualized on a JEM-2100 electron microscope (JEOL, Japan) and images were captured using an 11-megapixel Orius digital camera (Gatan, USA). For NTA, EV were analyzed using a NS500 Nanoparticle tracking instrument (NanoSight NTA 3.0 Nanoparticle Tracking and Analysis Release Version Build 0064). The instrument’s camera was set at level 15 and a threshold of three pixels.

For Western blotting, EV were diluted in Lamelli Buffer (Bio-Rad, Australia), separated on a mini TGX 8–16% gel (Bio-Rad, Australia). The Trans-Blot Turbo Transfer System (Bio-Rad, Australia) was used to transfer the proteins from the gel to the membrane which was then probed with the following primary antibodies: TSG-101 (1:1000, clone EPR7130B, Abcam), CD9 (1:500, clone MM2/57, Life-Technologies), Calnexin (1:300, clone 1563, Novus Biologicals, USA) and: secondary antibodies (sheep anti-mouse IgG-HRP conjugate, polyclonal, 1:2000, GE Healthcare, donkey anti-rabbit IgG-HRP conjugate, polyclonal, 1:2000, GE Healthcare).

### DNA extraction

DNA extraction was carried out as per method previously published by our group^39^. DNA was extracted using the QIAamp DNA Microkit (Qiagen, Australia) according to the manufacturers’ instructions. DNA concentration was quantified using the Qubit high-sensitivity dsDNA kit (Thermo Fisher Scientific, Australia). DNA quality of one patient (study ID #B-010) was also characterized using High Sensitivity DNA bioanalyzers (Agilent Technologies, Australia).

### Low-pass whole genome sequencing

Low-pass whole genome sequencing was performed at the Peter MacCallum Cancer Centre using a previously published method^18^. DNA was eluted in 50 μL of low TE buffer/nuclease-free water and quantified with QuBit high sensitivity (Thermo Fisher Scientific, Australia), and subsequently normalized to a volume of 50 μL in low TE buffer before sonication. DNA was sheared with sonication (Covaris S2 system) for 1 × 40 s, with the following parameters: duty cycles of 10, an intensity of five, and 200 cycles/burst.

Library preparation was performed with the NEBNext Ultra II DNA Library Preparation Kit (New England Biolabs, USA) as per manufacturer’s instructions, with minor modifications. To each 50 μL sample of DNA 3 μL of NEBNext Ultra II End Prep Enzyme Mix and 7 μL of NEBNext Ultra II End Prep Reaction Buffer was added, followed by a short thermal cycling run to carry out end repair, 5′ phosphorylation and 3′ dA-tailing (deoxyadenosine monophosphate-tailing). The thermocycling conditions used were; 30 min at 20 °C for one cycle (end repair stage), and 30 min at 65 °C for one cycle (adenylation or a-tailing stage), with a final hold of 4 °C. 1.5 μM of NEBNext Adaptors were then ligated to DNA with 60 μL of NEBNext Ultra II Ligation Master Mix and 1 μL of NEBNext Ligation Enhancer (1 μL) in a 30minute incubation at 20 °C. Adaptor loops were severed by treatment with USER Enzyme which removed the uracil binding the two halves of the loop together. For this step, DNA was incubated with 3 μL of enzyme for 15 min at 37 °C.

Clean-up of the adaptor-ligated DNA was performed using Agencourt AMPure XP Reagent (Beckman Coulter, Australia) as per section 3.8.1 of the manufacturer’s instructions (‘Library preparation’). The purified adaptor-ligated DNA was then enriched with 11 cycles of PCR. The cycling conditions were; 30 s at 98 °C for one cycle (initial DNA denaturation), 10 s at 88 °C (DNA denaturation) and 75 s at 65 °C (primer annealing and DNA extension) for 11 cycles, and five minutes at 65 °C for one cycle (final extension phase) prior to a final hold at 4 °C. For PCR, 25 μL of NEBNext Ultra II Q5 Master Mix was added to adaptor-ligated DNA, along with 5 μL each of forward (Universal Primer i5) and reverse (Index Primer i7) primers. PCR products were then purified using Agencourt AMPure XP Reagent, increasing the incubation times to 20 min and 10 min for beads and elution low TE buffer, respectively. Library fragment size distribution was determined with the Agilent 4200 TapeStation System (Agilent Technologies, Australia).

Libraries were normalized, pooled and diluted to a final concentration of 1.8 pM as per the NextSeq 500 High Output v2 Reagents Kit guide (Illumina, Australia). 1.3 mL of the diluted library pool was loaded onto a reagent cartridge and inserted into the Illumina NextSeq 500 System for low coverage sequencing, along with a flow cell and buffer cartridge. The 75 bp paired-end sequencing reaction was performed, resulting in an average of 50 million reads for each sample. Sequencing of those samples led to a genome coverage of 0.5–1 × per sample.

### CNA analysis

Raw reads were trimmed for sequencing adaptors using Trimgalore v0.4.5, a wrapper for Cutadapt v1.12 using default parameters. Trimmed reads were then aligned to the hg19 reference genome using BWA v0.7.15 with default parameters^46^. Alignment statistics and genome coverage metrics were extracted using GATK v3.7 and Picard v2.9.0. Segmentation analysis was performed using qDNASeq and the resultant output files were summarised using R, version 1.6.2^20^. Overlap analysis was performed using bedtools v2.17.0 and plotted with UpSetR v1.4.0 within R v3.6.0 and RStudio v1.2.1335-1.
