# Author Correction: Low-coverage whole-genome sequencing of extracellular vesicle-associated DNA in patients with metastatic cancer

**DOI:** 10.1038/s41598-021-94496-8

**Published:** 2021-07-27

**Authors:** Bella Nguyen, Nicholas C. Wong, Tim Semple, Michael Clark, Stephen Q. Wong, Connull Leslie, Bob Mirzai, Michael Millward, Katie Meehan, Annette M. Lim

**Affiliations:** 1 Department of Medical Oncology, Sir Charles Gairdner Hospital, Perth, WA Australia; 2 School of Biomedical Sciences, University of Western Australia, Perth, WA Australia; 3 Monash Bioinformatics Platform, Monash University, Victoria, Australia; 4 Department of Medical Oncology, Peter MacCallum Cancer Centre, Melbourne, VIC Australia; 5 Peter MacCallum Cancer Centre, Melbourne, VIC Australia; 6 School of Medical and Health Sciences, Edith Cowan University, Joondalup, WA Australia; 7 School of Pathology and Laboratory Medicine, The University of Western Australia, Perth, WA Australia; 8 Department of Anatomical Pathology, PathWest, QEII Medical Centre, Perth, WA Australia; 9 Department of Otorhinolaryngology, Head and Neck Surgery, Chinese University of Hong Kong, Shatin, Hong Kong; 10 School of Medicine, The University of Western Australia, Perth, WA Australia

Correction to: *Scientific Reports* 10.1038/s41598-021-83436-1, published online 17 February 2021

The original version of this Article contained errors in the Affiliations.

Annette M. Lim was incorrectly affiliated with ‘School of Medical and Health Sciences, Edith Cowan University, Joondalup, WA, Australia.’

In addition, Tim Semple and Stephen Q. Wong were incorrectly affiliated with ‘Department of Medical Oncology, Peter MacCallum Cancer Centre, Melbourne, Australia.’

Finally, Affiliation 4 and 5 were incorrectly given as ‘Department of Medical Oncology, Peter MacCallum Cancer Centre, Melbourne, Australia.’ and ‘Sir Peter MacCallum Department of Oncology, The University of Melbourne, Victoria, Australia.’

The correct affiliations are listed below:

Affiliation 4:

Department of Medical Oncology, Peter MacCallum Cancer Centre, Melbourne, VIC, Australia

Annette M. Lim

Affiliation 5:

Peter MacCallum Cancer Centre, Melbourne, VIC, Australia

Tim Semple and Stephen Q. Wong

The original Article has been corrected.

